# WF SS-OCTA for detecting diabetic retinopathy and evaluating the effect of photocoagulation on posterior vitreous detachment

**DOI:** 10.3389/fendo.2022.1029066

**Published:** 2022-12-02

**Authors:** Yi Gong, Liying Hu, Linni Wang, Yan Shao, Xiaorong Li

**Affiliations:** Tianjin Key Laboratory of Retinal Functions and Diseases, Tianjin International Joint Research and Development Centre of Ophthalmology and Vision Science, Eye Institute and School of Optometry, Tianjin Medical University Eye Hospital, Tianjin, China

**Keywords:** widefield swept source optical coherence tomography angiography, ultra-widefield fluorescein angiography, nonperfusion area, neovascularization, diabetic retinopathy, posterior vitreous detachment

## Abstract

**Purpose:**

This study aimed to assess the clinical usefulness of widefield swept source optical coherence tomography angiography (WF SS-OCTA) for detecting microvasculature lesions in diabetic retinopathy (DR) by comparing it with ultra-widefield fluorescein angiography (UWFFA) and to investigate the effect of panretinal photocoagulation (PRP) on posterior vitreous detachment (PVD) status.

**Methods:**

Patients with severe non-proliferative DR (NPDR) or proliferative DR (PDR) who were initially treated with PRP were enrolled. They underwent WF SS-OCTA with a 12×12-mm scan pattern of five visual fixations at baseline and at least a 3-month follow-up after PRP treatment. Patients with no contraindications underwent imaging with UWFFA within a week. Images were evaluated using two methods for the areas of the visible field of view (FOV), non-perfusion area (NPA), presence of neovascularization of the disc (NVD), neovascularization elsewhere (NVE), and PVD status.

**Results:**

In total, 44 eyes of 28 patients with DR that were initially treated with PRP were analyzed. The FOV of the UWFFA was significantly wider than that of the WF SS-OCTA. The quantitative measurement of the NPAs was consistent between the two methods. NPAs more than 5DA outside the panoramic OCTA imaging area were detected in 1 eye with NPDR (8.3%) and in 10 eyes with PDR (47.8%). WF SS-OCTA had high detection rates for NVDs and NVEs, with a low rate of false positives. After PRP treatment, no eyes indicated progression in the PVD stages around the macula, optical disc, or NVEs at the short follow-up.

**Conclusion:**

WF SS-OCTA is clinically useful for evaluating NPAs and neovascularization in DR. PRP treatment does not induce PVD development in the short term.

## Introduction

According to reports from the International Diabetes Federation, 537 million adults had diabetes in 2021, and this number is predicted to increase to 784 million by 2045 (1). Diabetic retinopathy (DR) is a severe microvascular complication of diabetes characterized by microaneurysms, intraretinal microvascular abnormalities, venous beading, non-perfusion area (NPA), and neovascularization (NV). The diagnosis and treatment of DR are mainly based on fundus photography (including ETDRS 7-standard field 35 mm stereoscopic color 30° fundus photographs and ultra-widefield fundus [UWF] photography), fluorescein angiography (FA), and optical coherence tomography (OCT) (2–4).

FA is currently the gold standard for the clinical evaluation of retinal vascular features in DR. However, as an invasive examination, it has some contraindications, such as renal insufficiency, cardiovascular diseases, and possible risks ranging from nausea and vomiting to anaphylaxis to even death. According to the American Academy of Ophthalmology Preferred Practice Patterns, FA is not a routine examination for patients with diabetes and is not indicated for monitoring the therapeutic effect or progression of DR (5).

OCTA is a noninvasive imaging technique for evaluating vasculature circulation in the choroid and any layer of the retina (6). It can also provide objective information on the vitreoretinal interface (VRI) simultaneously, such as posterior vitreous detachment (PVD), which is important for the growth of NVs. Due to the limited field of view (FOV), conventional OCTA (3 × 3 mm or 6 × 6 mm) only assesses the macular vascular network qualitatively and quantitatively and is meaningful in the evaluation of macular pathologies (6, 7).

Meanwhile, the commercial widefield swept source OCTA (WF SS-OCTA) system (VG200, SVision Imaging, Ltd., Luoyang, China) can capture a 12 × 12-mm angiography image in a single scan. Additionally, with Flexible Montage TM technology, the FOV further expands to 80° × 60° (23.5 × 17.5 mm).

WF SS-OCTA can obtain high-resolution images of the VRI from the macula to the mid-peripheral retina, which is wider than conventional OCT/OCTA. According to previous studies, PVD stage may be related to the pathophysiology of diabetic macula edema (DME) and NVs (8, 9). Thus, prophylactic induction of PVD may benefit patients with DR.

In this study, we explored whether WF SS-OCTA could substitute UWFFA in clinical practice to evaluate the DR lesions and observed the short-term effect of panretinal photocoagulation (PRP) treatment on PVD progression.

## Materials and methods

### Study participants

This observational study was conducted at Tianjin Medical University Eye Hospital between September 2020 and February 2022 and followed the tenets of the Declaration of Helsinki. Written informed consent was obtained from all patients.

The inclusion criteria were as follows: age ≥20 years, confirmed diagnosis of type 1 or type 2 diabetes mellitus, severe non-proliferative DR (NPDR) or proliferative DR (PDR) that required PRP treatment, no previous history of intravitreal injection. Meanwhile, the exclusion criteria were as follows: history of fundus laser treatment, presence of other retinal diseases, history of glaucoma, media opacities such as cataract or severe vitreous hemorrhage, and underwent cataract surgery during the study.

### Study protocol

The enrolled patients underwent comprehensive ophthalmological examinations, including measurement of visual acuity and intraocular pressure, slit-lamp biomicroscopy, UWFFA (except for patients with contraindications), WF SS-OCTA at baseline, and a 3-month follow-up after PRP.

UWFFA images were obtained after standard intravenous injection of 5 ml of 10% sodium fluorescein using the Optos Optomap Panoramic 200Tx imaging system (Optos, PLC, Dunfermline, Scotland), which theoretically covered nearly the entire retina (up to 200°). The configurations of retinal vessels and hyperfluorescent areas were evaluated in the early phase to reduce the effect of dye leakage.

Five OCTA en-face images of 12×12-mm regions (center, temporal superior, temporal inferior, nasal superior, and nasal inferior) were acquired using the WF SS-OCTA system (VG200, SVision Imaging, Ltd., Luoyang, China). This instrument was equipped with a swept-source laser with a central wavelength of 1050 nm and operated at a scanning rate of 200,000 A-scans per second. The axial resolution in the tissue and lateral resolution at the retinal surface were 5 μm and 20 μm, respectively. Each image was obtained using a raster scan protocol of 1024 B-scan positions per volume, two repeated B-scans per B-scan position, and 1024 A-scans per B-scan. The system was equipped with an artificial intelligence-assisted tracking system to eliminate eye motion artifacts and retain the original blood vessel signals using the SS-PAR algorithm. In the case of segmentation errors, the segmentation of the different layers was manually corrected. The WF SS-OCTA en face montage was generated automatically (up to 80°×60°), and only high-quality images were included in our study (signal strength≥6).

Data on age, sex, and stage of DR were obtained from medical records.

### Treatment protocol

A 577-nm pattern scan laser photocoagulator was used for PRP. Parameters (power × duration) were determined by visual observation; the dose was considered adequate if the spot turned gray-white immediately after the laser. In total, 1200–1600 spots (300–500-μm diameter for each spot) were delivered in three sessions. Intravitreal anti-vascular endothelial growth factor (anti-VEGF) therapy was added if DME was present.

### Image processing and analysis

NPAs were defined as the complete absence of retinal capillaries with dark or gray areas on both UWFFA and OCTA. NVs were characterized by abnormal vessels that grew towards the vitreous cavity. The retinal vasculature slab and VRI slab of OCTA were used to detect NVs combined with B-scan images for further confirmation (10). Neovascularization of the disc (NVD) was defined as a lesion located in the disc or within 1-disc diameter from the margin, whereas neovascularization elsewhere (NVE) was located outside this area (11).

PVD stage and progression were classified according to the study by Itakura and Kishi (12, 13) as follows: stage 0 (no PVD), stage 1 (paramacular PVD), stage 2 (perifoveal PVD), stage 3 (vitreofoveal separation and peripapillary PVD), and stage 4 (complete PVD).

**Figure 1:**
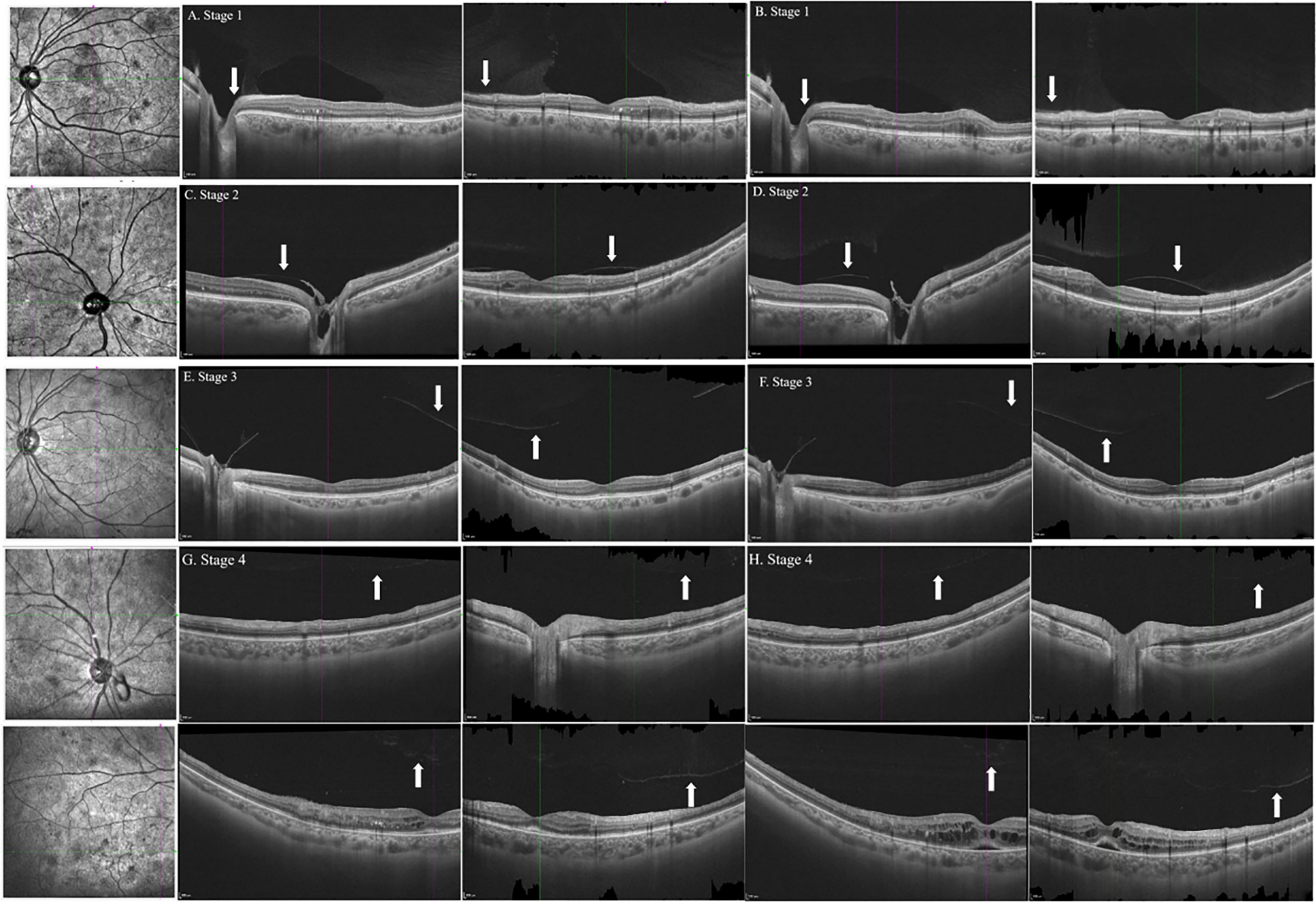
B-scan images of horizontal and vertical cross-sections of representative eyes with the stages 1, 2, 3, and 4 PVD in patients with DR at baseline **(A, C, E, G)** and follow-up **(B, D, F, H)**. Arrows indicated the posterior vitreous. PVD, posterior vitreous detachment; DR, diabetic retinopathy.

**Figure 2:**
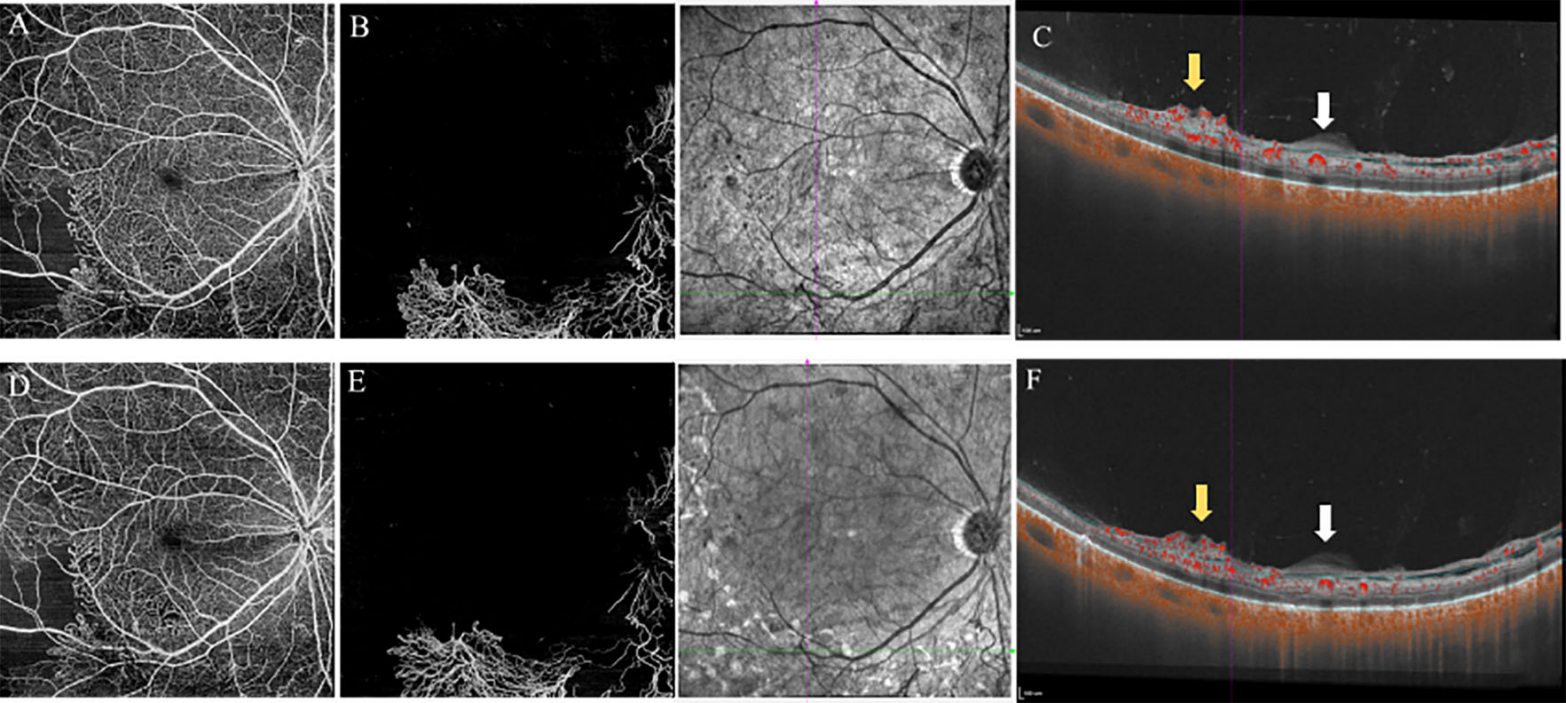
12×12mm SS-OCTA slabs of the retina **(A, D)**, VRI **(B, E)**, and B-scan images **(C, F)** showed features of PDR eyes at baseline (top) and 3-month follow-up (bottom). The area of NVs was reduced from 14.77mm^2^ **(B)** to 13.11mm^2^ **(E)** after PRP treatment (measured by Angiotool, Version 0.6a). B-scan images revealed no PVD around the location of NVs at baseline and after PRP treatment. White arrows indicated the thickened posterior vitreous (PVC). Yellow arrows indicated the NVs. VRI, Vitreoretinal Interface; PVD, posterior vitreous detachment; NVs, Neovascularization; PRP, panretinal photocoagulation.

The disc area (DA), FOV, NPAs, and horizontal and vertical lengths in WF SS-OCTA and UWFFA images were measured manually and separately using the ImageJ software (Version 1.53c). The values of FOV/DA and each NPA/DA in the two different widefield devices were calculated and compared. The PVD status around the macula, optical disc, and NVEs at baseline and at the 3-month follow-up after PRP treatment was recorded (**Figures 1**, **2**).

### Statistical analysis

Statistical analyses were performed using Statistical Packages for Social Sciences V.21.0 (SPSS V.21.0) and MedCalc (Version 19.4). Continuous variables are expressed as mean values ± standard deviation or median and interquartile range. Data were analyzed using the Kolmogorov–Smirnov test or Shapiro–Wilk test to evaluate the normality of the sample distribution. The agreement of NPAs measured by WF SS-OCTA and UWFFA was assessed using Bland–Altman analysis. The sensitivity and specificity of WF SS-OCTA were calculated based on UWFFA images as the reference standard for evaluating NVD and NVE. Agreement of measurements between readers was assessed using interclass correlation coefficient (ICC).

## Results

### Patient characteristics

In total, 44 eyes of 28 patients with DR were analyzed. Only seven eyes of five patients were subjected to WF SS-OCTA imaging due to contraindications for UWFFA; the DA in two eyes of two patients could not be measured on UWFFA due to leakage of the NVD. Five eyes of three patients were excluded because of media opacities or poor fixation ability. The patient characteristics are described in **Table 1**.

**Table 1:** Clinical characteristics.

Parameter	Values
Eyes/patients	44/28
Age, Mean ± SD, y	51.43 ± 10.93
Sex	
Male	19
Female	9
DR severity	
NPDR PDR	14 30

### Quantitative measurement of the FOV

**Figure 3:**
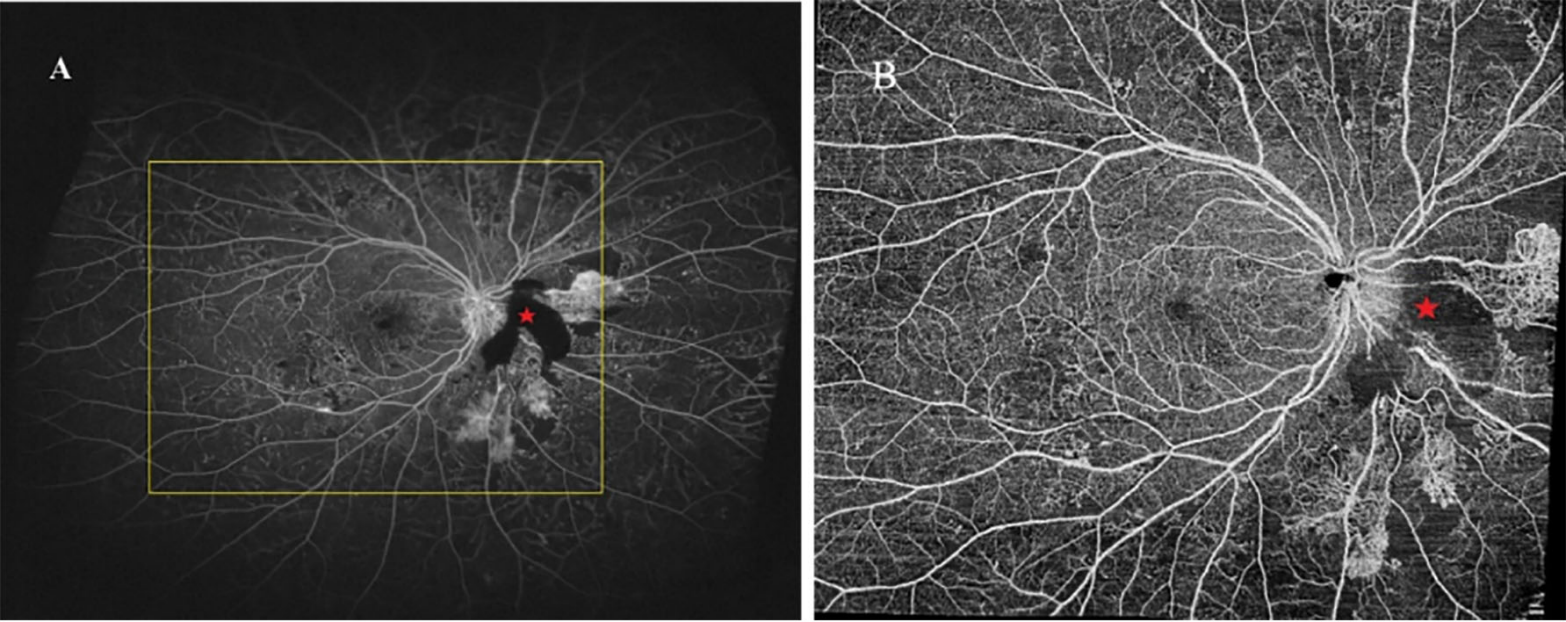
UWFFA **(A)** and WF SS-OCTA **(B)** posterior pole montage of a representative eye. UWFFA performed by Optos Optomap Panoramic 200Tx imaging system with the yellow square indicated the same FOV of WF SS-OCTA obtained by VG200, SVision Imaging system. The area noted with red pentagram was caused by funds hemorrhage.

The images of the UWFFA and WF SS-OCTA en face montage are presented in **Figure 3**. The mean extension ratios of the horizontal and vertical field to the corresponding diameter of the optic disc were 11.13 ± 0.97 (range, 8.47–12.47), 9.40 ± 0.86 (range, 7.16–10.64) in WF SS-OCTA, and 18.79 ± 2.64 (range, 12.87–23.05) and 10.88 ± 1.63 (range, 7.91–14.70) in UWFFA. The ratios of the horizontal and vertical dimensions between WF SS-OCTA and UWFFA were significantly different (P<0.05). The mean FOV to DA in the two methods were 118.28 ± 19.70 and 209.95 ± 37.62, respectively, and a significant difference in FOV/DA values was observed (P<0.001).

### Quantitative measurement of NPAs

#### Within the area of the panoramic WF SS-OCTA image

**Figure 4:**
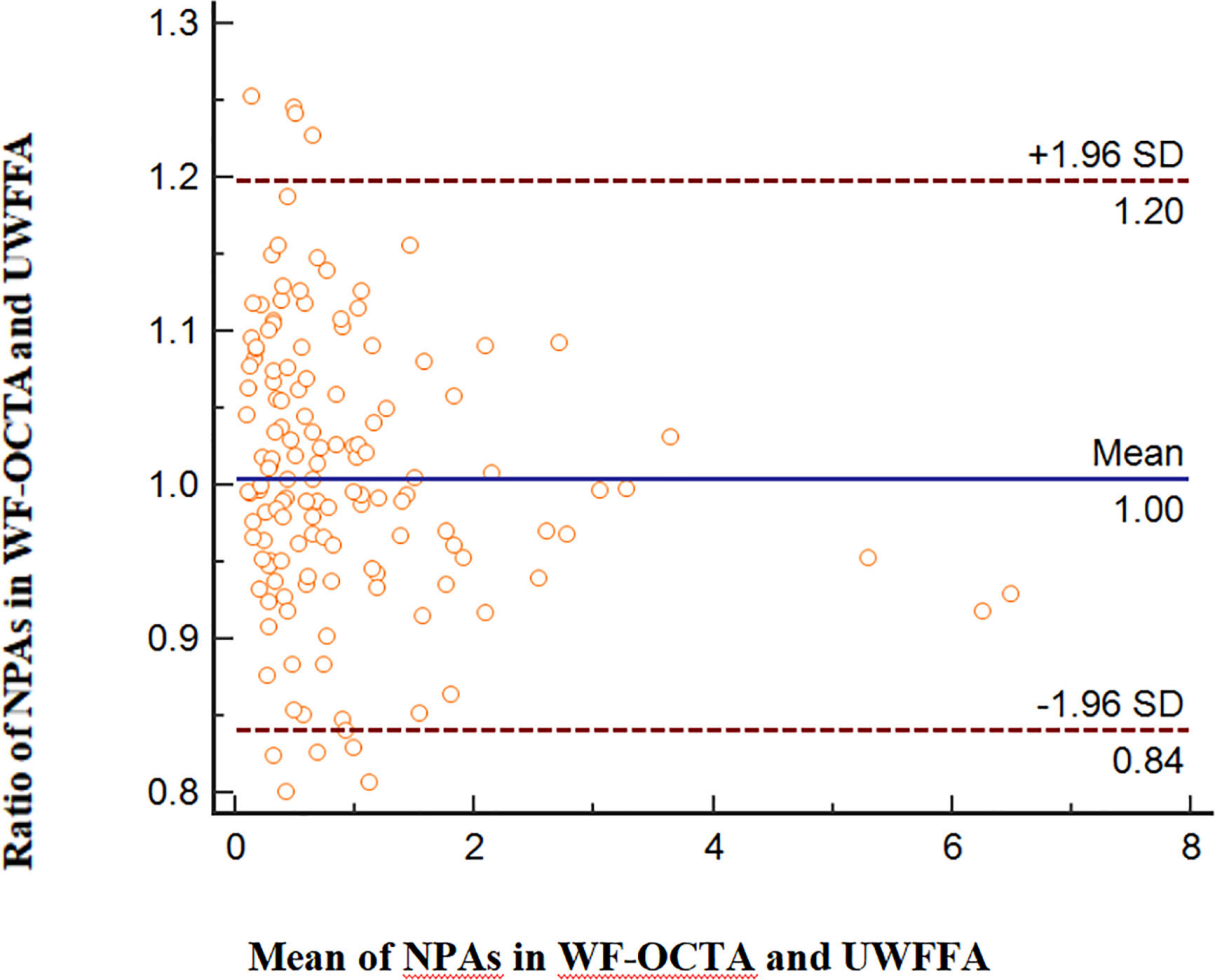
Comparison of NPAs in WF SS-OCTA and UWFFA *via* the Bland–Altman approach, displaying bias and the 95% LoA.

The ratios of NPA/DA in the retinal layer measured using WF SS-OCTA and UWFFA were normally distributed (P=0.413). **Figure 4** presents Bland–Altman plots for NPAs. The horizontal lines represent the mean ratio and 95% confidence interval limits. According to the Bland–Altman method, the mean ratio of the NPAs measured by WF SS-OCTA to UWFFA was 1.00. Here, 132 of 141 paired values (93.62%) were situated within the 95% level of agreement (LoA, 0.84–1.20). The interobserver ICC was 0.890 (95% CI: 0.850–0.920).

#### Outside the area of the panoramic WF SS-OCTA image

Outside the area of the panoramic OCTA images, the NPA/DA values detected by UWFFA ranged from 0 to 46.67. Of the 35 eyes measured in our study, NPA (>5 DA) was observed in 1 eye (8.3%) in patients with NPDR and in 11 eyes (47.8%) in patients with PDR (**Table 2**). The interobserver ICC was 0.994 (95% CI: 0.988–0.997).

**Table 2:** NPAs measured by UWFFA out of the area of the panoramic OCTA image.

Features (NPAs/DA)	Number (%)
NPDR ≤5 5-10 ≥10	11 (91.7%) 1 (8.3%) 0 (0%)
PDR ≤5 5-10 ≥10	12 (52.2%) 3 (13.0%) 8 (34.8%)

NPAs, Nonperfusion areas; DA, Disc area; NPDR, Nonperfusion proliferative diabetic retinopathy; PDR, proliferative diabetic retinopathy.

#### Detection of NVs

Altogether, 25 eyes with PDR were analyzed in this study. NVDs were detected in nine eyes using UWFFA. The sensitivity and specificity of WF SS-OCTA for NVD detection were 100% and 96.67%, respectively. The ability to accurately identify NVEs was also assessed. 96 NVEs were detected by WF SS-OCTA, of which 94 NVEs were confirmed by UWFFA, two of which may be false positives (FP, 2.2%) because of no evident leakage in the late frames of UWFFA (**Table 3**). Additionally, 21 NVE lesions were identified using UWFFA in the area of WF SS-OCTA (18.3%), whereas no eyes only existed NVs in this area.

**Table 3:** Detection rates of NVEs in proliferative diabetic retinopathy.

	NVE lesions	Confirmed(%)	Not confirmed(%, FP)
UWFFA	94	Reference	Reference
WF SS-OCTA	96	94(100%)	2(2.2%)

UWFFA, ultra-widefield fluorescein angiography; WF SS-OCTA, widefield swept source optical coherence tomography angiography; NVE, neovascularization elsewhere; FP, False Positive

#### PVD status

Altogether, 20 eyes of 14 patients completed the follow-up, and the median time was 106.0 ± 30.0 days (range: 80–187 days). Owing to DME, three eyes of two patients received three monthly injections of anti-VEGF agents, except for PRP treatment during the investigation. At baseline, 19 eyes had vitreoretinal relationships at stages 1 (50.0%), 2 (35.0%), and 3 (10.0%). Stage 4 vitreoretinal relationship (complete PVD) was observed in only one eye (5.0%) (**Table 4**). B-scan images of all the eyes revealed no PVD at the NV locations. However, after PRP treatment, PVD around the macula and optical disc did not develop in any of the eyes (20/20, 100%; **Figure 1**). No evident progress in PVD around the NVs was observed (15/15, 100%; **Figure 2**).

**Table 4:** PVD stages at baseline.

Stages	Number (%)
Stage 0 Stage 1 Stage 2	0 10 (50.0%) 7 (35.0%)
Stage 3	2 (10.0%)
Stage 4	1 (5.0%)

PVD, posterior vitreous detachment.

## Discussion

This study compared the clinical utility of WF SS-OCTA and UWFFA for detecting DR lesions. The distribution of diabetic microangiopathy is widely known to be non-uniform within the retina (14, 15), and identifying lesions in the perifoveal and periphery is crucial for evaluating the risk of DR (16). In this study, we calculated the FOV captured by WF SS-OCTA and UWFFA. Although WF SS-OCTA had already expanded the imaging area substantially compared with conventional models of 3 mm × 3 mm and 6 mm × 6 mm, WF SS-OCTA still captured smaller areas of the fundus than UWFFA (P<0.001), and the ratios in both horizontal and vertical dimensions were significantly different (P<0.05).

Our results accord with those of previous studies demonstrating that OCTA had high sensitivity and specificity in detecting NPAs and NVs, which are crucial for evaluating DR progression and courses of treatment (10, 17–20). As is well established, NPAs represent retinal or macular ischemia, which is associated with DME and NVs. A previous study has reported that peripheral NPAs were significantly higher in the eyes with PDR than in the eyes with NPDR, whereas NPAs in the posterior pole were not significantly different (21). In this study, we conducted a quantitative assessment of NPAs within and outside WF SS-OCTA images using two checking devices. The Bland–Altman consistency analysis revealed that the points out of 95% LoA were 9 out of 141 (6.38%); therefore, we considered that the consistency for NPAs between WF SS-OCTA and UWFFA was good. However, in our study, out of 35 eyes detected by UWFFA and WF SS-OCTA, NPAs (>5 DA) outside the panoramic OCTA image area were detected in one eye with NPDR (8.3%) and in 11 eyes with PDR (47.8%). Despite the limited number of patients in our study, WF SS-OCTA still had some limitations in scanning peripheral NPAs compared with UWFFA, especially in PDR. The implications of far-peripheral lesions on DR severity and progression over time remain unknown. Some recent studies have reported that the far peripheral retina beyond the FOV of the current WF SS-OCTA instruments did not significantly contribute to the diagnosis and management of these patients (22–24). A possible clinical use for identifying peripheral NPAs is to target PRP in these regions, although no evidence can support that this approach could decrease the anti-VEGF injection burden or improve vision outcomes (25). Hence, the utility of quantifying NPAs in clinical practice requires further investigation.

Compared to NPAs, the meaning of determining and monitoring NVs is well known. In this observational study, WF SS-OCTA has a sensitivity and specificity for detecting NVDs of 100% and 96.67%, respectively, and a high detection rate for NVEs (100%) with a low rate of false positives (2.2%). WF SS-OCTA might be clinically adequate for identifying NVs in PDR cases as most NVs were observed within the mid-periphery of the retina, which were covered by WF SS-OCTA images (19).

In recent years, the absence of PVD and presence of an intact VRI have become generally considered essential for the growth of NVs in DR (26, 27). Therefore, identifying treatments to liquefy the vitreous gel and weaken vitreoretinal adhesions may be helpful for patients with diabetes. Prophylactic induction of PVD before the onset of NVs may be effective for preventing PDR progression. However, few studies have reported the frequency of PVD following intravitreal injection. Geck et al. observed a 25% PVD rate after injections within a mean follow-up of 11.1 weeks (28). Özsaygili et al. have reported that PVD occurred in approximately 18 % of the DME cohort during three aflibercept injections (29). However, Veloso et al. have reported PVD in only 7 of 125 eyes after a follow-up of 21.1 months (30).

Moreover, according to previous clinical studies using biomicroscopy, the incidence of PVD was higher in patients with DR who received PRP treatment than in those who did not (the mean follow-up times were 4 years and 4.5 years, respectively) (31, 32). Thus, PRP may induce PVD and provide therapeutic benefits. Moreover, the areas of PVD could serve as strategic locations for pars plana vitrectomy to delaminate fibrovascular membranes (FVM) (33). Therefore, prior to surgery for PDR, according to clinical experience, some vitreoretinal surgeons intend to perform PRP treatment, which might induce PVD progression to avoid exerting undue traction on the retina, complications of iatrogenic retinal break, and inadvertent transection of active FVM. However, our results differed from previous conclusions. To the best of our knowledge, no studies have used OCT/OCTA to evaluate the effects of PRP on inducing PVD in patients with DR. In the present study, we observed no significant change in PVD status around the macula, optical disc, and NVs after PRP treatment in the short term. The therapeutic benefit of PRP in regressing retinal neovascularization might not be achieved by mediating the occurrence of PVD. Our conclusions being different from those of Sebag et al. may be attributed to the follow-up time and patient heterogeneity.

The limitations of this study include the small sample size, short follow-up period, and heterogeneity in the PRP parameters. We also measured NPAs manually, which is impractical for clinical use. With the development of technology and deep learning, a wider imaging mode and more accurate automated measurement in OCTA may be available in the future. Additionally, eyelash artifacts prevented clear imaging of the inferior far retinal periphery, which might have overestimated the extent of the non-perfusion areas (34). To avoid eyelash artifacts, tape fixation and the examiner’s assistance with cotton swabs were used. However, this might decrease the efficiency of the examination and patient comfort during the examination. Finally, the effect of PRP on PVD is complicated by anti-VEGF injections in eyes with DME because the injections may have a role in inducing PVD. To definitively answer the question of whether, how, and when PRP affects PVD progression, a large, long-term, prospective study utilizing WF SS-OCT/OCTA acquisition protocols is needed.

## Conclusion

Overall, WF SS-OCTA was consistent with UWFFA in evaluating NPAs and NVs in DR. However, it still had limitations in terms of clinical value because of the area. PRP treatment could not change the PVD stages around the macula, optical disc, and NVs in patients with DR in the short term.

## Data availability statement

The raw data supporting the conclusions of this article will be made available by the authors, without undue reservation.

## Ethics statement

The studies involving human participants were reviewed and approved by Ethics Committee of Tianjin Medical University Eye Hospital. The patients/participants provided their written informed consent to participate in this study. Written informed consent was obtained from the individual(s) for the publication of any potentially identifiable images or data included in this article.

## Author contributions

YG and LH were responsible for drafting the manuscript, as well as the acquisition, analysis and interpretation of data. LW collected and interpreted the data. YS and XL contributed to the conception and design of the current study. All authors contributed to the article and approved the submitted version.

## Funding

National Natural Science Foundation Project (81900891), Global Ophthalmology Awards Program 2020 (482667), Key Project of Internet Cross-Border Integration Innovation and Technology of Tianjin (18ZXRHSY00210), Tianjin Clinical Key Discipline Construction Project (TJLCZDXKT004), Tianjin Clinical Key Discipline Construction Project (TJLCZDXKQ015), Science and Technology Project of Tianjin Binhai New District Health Commission (2019BWKY023).

## Acknowledgements

We thank all patients for their cooperation in this study.

## Conflict of interest

The authors declare that the research was conducted in the absence of any commercial or financial relationships that could be construed as a potential conflict of interest.

## Publisher’s note

All claims expressed in this article are solely those of the authors and do not necessarily represent those of their affiliated organizations, or those of the publisher, the editors and the reviewers. Any product that may be evaluated in this article, or claim that may be made by its manufacturer, is not guaranteed or endorsed by the publisher.
